# Authors’ reply: Two severe human cases due to swine influenza A (H1N1)v in October 2016 in Europe were chronologic coincident yet distinct events

**DOI:** 10.2807/1560-7917.ES.2017.22.10.30480

**Published:** 2017-03-09

**Authors:** Francesca Rovida, Antonio Piralla, Federico Capra Marzani, Ana Moreno, Giulia Campanini, Francesco Mojoli, Marco Pozzi, Alessia Girello, Chiara Chiapponi, Fausto Vezzoli, Paola Prati, Elena Percivalle, Anna Pavan, Maria Gramegna, Giorgio Antonio Iotti, Fausto Baldanti

**Affiliations:** 1 SS Virologia Molecolare, SC Microbiologia e Virologia, Fondazione IRCCS Policlinico San Matteo, Pavia, Italy; 2 Anestesia e Rianimazione, Dipartimento di Emergenza ed Urgenza, Fondazione IRCCS Policlinico S. Matteo, Pavia, Italy; 3 Istituto Zooprofilattico Sperimentale della Lombardia ed Emilia Romagna, Brescia, Italy; 4 Unità di Anestesia, Rianimazione e Terapia Antalgica, Dipartimento di Scienze Clinico-Chirurgiche, Diagnostiche e Pediatriche, Università degli Studi di Pavia, Pavia, Italy; 5 Istituto Zooprofilattico Sperimentale della Lombardia ed Emilia Romagna, Parma, Italy; 6 Istituto Zooprofilattico Sperimentale della Lombardia ed Emilia Romagna, Lodi, Italy; 7 Istituto Zooprofilattico Sperimentale della Lombardia ed Emilia Romagna, Pavia, Italy; 8 Agenzia di Tutela della Salute, Pavia, Italy; 9 Direzione Generale Sanità, Regione Lombardia, Milano, Italy; 10 Dipartimento di Scienze Clinico-Chirurgiche, Diagnostiche e Pediatriche, Università degli Studi di Pavia, Pavia, Italy

**Keywords:** zoonotic infections, respiratory infections, influenza virus

**To the editor**: Indeed, the similarities between the cases reported by Fraaij et al. from the Netherlands [1] and our group in Italy [2] are somewhat striking. Both cases occurred in October 2016, both cases presented with severe respiratory syndrome and in both cases, a swine influenza virus (SIV) strain circulating in a nearby pig farm was detected in the patient. In the paper by Fraaij et al., the patient had visited a pig farm, but not had direct contact with pigs [1]. In our paper, the patient stated that he had no contact with infected pigs, but his brother worked on a pig farm. Although the data presented in our paper strictly adhere with official reports [2], we also agree that unreported visits to a pig farm or direct contact with infected pigs through contaminated surfaces or via aerosol are possibilities that cannot be excluded. However, a major difference between the Dutch and Italian case were the SIV strains recovered from the patients, indicating the occurrence of chronologically coincidental yet distinct events.

We share all the concerns raised by the Authors of the Letter to the editor [3]. In particular: (i) attention to severe zoonotic influenza A infections in humans should be as high in Europe as in any other region of the world. (ii) Both reported cases occurred earlier than the influenza season in humans and this event may have had an impact on established surveillance procedures and reporting which are activated at the beginning of the season. (iii) Virus whole genome sequencing should be the gold standard for specimens with inconclusive results or nontypeable influenza strains (indeed, this approach was followed by both the Dutch and Italian groups). (iv) We strongly recommend the inclusion of pan-influenza A molecular assays in the work-up of all patients with severe respiratory syndromes, irrespective of seasonality. (v) We fully support the need for unrestricted sharing of biological materials as well as epidemiological, clinical and sequence data.

In addition, we support the suggestion for follow-up investigations in patients with a documented SIV infection, a task that might not be easy to achieve. As far as our recent experience is concerned, at a follow-up telephone visit on 7 February 2017 [2]. As confirmed by his brother, the former patient was well, but not available for further questions.

Finally, we would like to emphasise that swine influenza monitoring programmes at the Istituto Zooprofilattico Sperimentale della Lombardia e dell'Emilia Romagna (IZSLER) have been in place since the late 1990s, especially in northern Italy where more than 75% of the Italian swine industry is located. These programmes (further improved since 2009) are mainly based on genome detection, virus isolation and sequencing of all respiratory forms and revealing continuous circulation of H1N1, H3N2 and H1N2 SIVs as well as the isolation of influenza A(H1N1)pdm09 viruses in pigs.

Moreover, surveillance of SIV circulating in European pigs is carried out in many countries, mainly in western Europe, involving the networks *European Surveillance Network for Influenza in Pigs* (ESNIP 1, 2 and 3) which are aimed at expanding our knowledge on European SIV epidemiology [4]. However, judging by the small number of available SIV sequences, SIV surveillance appears to be less rigorous and systematic in other parts of Europe.
